# Monitoring Winter and Summer Abundance of Cetaceans in the Pelagos Sanctuary (Northwestern Mediterranean Sea) Through Aerial Surveys

**DOI:** 10.1371/journal.pone.0022878

**Published:** 2011-07-29

**Authors:** Simone Panigada, Giancarlo Lauriano, Louise Burt, Nino Pierantonio, Greg Donovan

**Affiliations:** 1 Tethys Research Institute, Milano, Italy; 2 Institute for Environmental Protection and Research - ISPRA, Roma, Italy; 3 RUWPA, University of St. Andrews, Scotland, United Kingdom; 4 International Whaling Commission, The Red House, Cambridge, United Kingdom; University of Western Australia, Zimbabwe

## Abstract

Systematic long-term monitoring of abundance is essential to inform conservation measures and evaluate their effectiveness. To instigate such work in the Pelagos Sanctuary in the Mediterranean, two aerial surveys were conducted in winter and summer 2009. A total of 467 (131 in winter, 336 in summer) sightings of 7 species was made. Sample sizes were sufficient to estimate abundance of fin whales in summer (148; 95% CI = 87–254) and striped dolphins in winter (19,462; 95% CI = 12 939–29 273) and in summer (38 488; 95% CI = 27 447–53 968). Numbers of animals within the Sanctuary are significantly higher in summer, when human activities and thus potential population level impacts are highest. Comparisons with data from past shipboard surveys suggest an appreciable decrease in fin whales within the Sanctuary area and an appreciable increase in striped dolphins. Aerial surveys proved to be more efficient than ship surveys, allowing more robust estimates, with smaller CIs and CVs. These results provide essential baseline data for this marine protected area and continued regular surveys will allow the effectiveness of the MPA in terms of cetacean conservation to be evaluated and inform future management measures. The collected data may also be crucial in assessing whether ship strikes, one of the main causes of death for fin whales in the Mediterranean, are affecting the Mediterranean population.

## Introduction

In view of the ‘unusually high’ abundance of cetaceans when compared with neighbouring areas and the high levels of human activities that may have a negative impact on cetaceans (and other species), Italy, France and the Principality of Monaco established the International Sanctuary for the Protection of Mediterranean Marine Mammals (hereafter ‘Pelagos Sanctuary’); it represents the world's first International High Seas Marine Protected Area and was incorporated in the list of Specially Protected Areas of Mediterranean Interest (SPAMIs) within the framework of the Barcelona Convention in 2001 [1].

**Figure 1:**
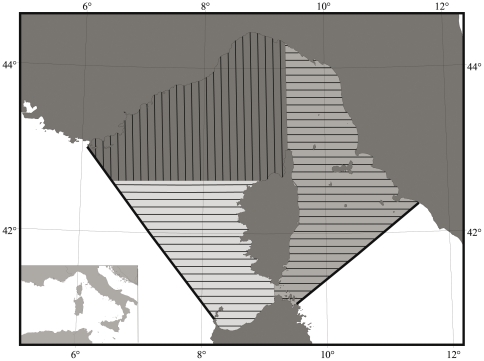
Map of the study area. The study area has been subdivided into three strata; the Pelagos Sanctuary borders and the 2000 m isobath are shown.

The Sanctuary, which includes the Ligurian Sea and portions of the Corsican and Tyrrhenian Seas (Fig. 1), encompasses a surface of ∼90 000 km^2^ and represents one of the most highly variable ecosystems throughout the Mediterranean Basin [2]. The need for focussed scientific research in order to provide a sound basis for managing human activities to maintain and improve the population status of cetaceans within the Sanctuary and in the wider Mediterranean is well-known, as is the need to enforce existing national and international regulations (e.g. see review by [3]). While the Pelagos Sanctuary represents a unique example and opportunity for marine conservation in the Mediterranean, without strong leadership and action, the risk of failure is ever-increasing [1].

Without good information on abundance, trends and population structure, it will not be possible to evaluate and mitigate for the known and potential threats to cetaceans within the Sanctuary, including ship strikes for fin whales (*Balaenoptera physalus*) and incidental mortality in legal and illegal fishing gear for striped dolphins (*Stenella coeruleoalba*) and other cetaceans [3]–[5], as well as habitat degradation and the possible effects of climate change [4], [6]–[8].

Fin whales and striped dolphins are probably the most abundant cetacean species in the Pelagos Sanctuary and, indeed, the Mediterranean as a whole [3]. Both species appear to be genetically separate from North Atlantic populations, with limited gene flow across the Strait of Gibraltar [9]–[12].

Although some abundance estimates exist [13], information on presence, distribution and abundance of cetaceans throughout the year is scattered and incomplete (see Appendix S1); data are particularly scarce during the winter months due to poor weather conditions for surveys. There are no abundance estimates for the eastern Mediterranean Sea.

Systematic monitoring of density and abundance of cetaceans is essential to inform conservation measures [14]. Although this is recognised in the Pelagos Sanctuary management plan, as well as international agreements such as the Agreement on the Conservation of Cetaceans in the Black Sea, Mediterranean Sea and Contiguous Atlantic Area (ACCOBAMS), to date there are few, if any, systematic monitoring programmes in place in the Mediterranean.

To begin to remedy this, the Italian Ministry of the Environment is funding an aerial survey programme and this paper presents estimates of abundance from the first two surveys that covered the complete Pelagos Sanctuary in winter and summer 2009.

## Methods

### Survey design and data collection

The survey was designed such that ‘distance sampling’ methods could be used to estimate abundance [15], [16]. The study area was subdivided into three strata (Fig. 1), following a bathymetric criteria and the available knowledge of cetacean presence and distribution. A total of 82 parallel line transects, 10 km apart, with a random start point were determined using the program Distance ver. 5.0 (http://www.ruwpa.st-and.ac.uk/distance/), to allow for homogeneous coverage probability over the selected area. Transects were oriented east-west in strata A and B and north-south in stratum C, to abut the coast line perpendicularly.

Both surveys were conducted using a *Partenavia P-68* aircraft, equipped with bubble windows to enable a full view of the trackline. The same three scientists were present on both surveys, at any one time, two acted as observers in the rear seats and a third acted as data recorder in the co-pilot seat; scientists changed seats at each landing to reduce fatigue. Survey height was 750 feet (229 m) which has been established as an optimum altitude for surveys targeting both large and small cetaceans [17]. Survey speed was around 100 kts (185 km/hr), balancing the need to fly as slow as possible to optimise sightings and the stall speed of the aircraft. Data collection was based on the protocol and software used for the aerial survey component of the SCANS-II programme [18].

Declination angle to sightings was measured with hand-held *Suunto* clinometers that, together with the aircraft altitude, provided a precise measurement of the perpendicular distance to the animal or group of animals. Environmental conditions (sea state, glare, cloud cover, turbidity and a subjective assessment of overall conditions) were recorded at the beginning of each transect and whenever a change occurred.

When on the trackline under acceptable conditions (sea state <4 on the Beaufort scale, estimated visibility >750 m), observers searched a 90° arc from abeam to ahead and from immediately below the plane outwards. When a sighting was made, the following data were recorded: declination angle to the sighting when it was abeam (or estimated to be abeam if the animal had dived), species, group size, initial cue, estimated swim direction, behaviour, and observer making the sighting. Only if there was some uncertainty in species identification or group size did the plane divert from the trackline to investigate and confirm the information; such activity was considered ‘off effort’. Whenever large groups of dolphins were sighted, they were relocated during ‘off effort’ operations to confirm group size; digital pictures of the whole group were taken and animals were counted and school size estimated *a posteriori*. Photographs were also used to assist with species identification. If small groups of dolphins were lost ‘off effort’ when they were attempted to be relocated for species checking, they were considered as un-identified small dolphins and not used in abundance analysis. In fact, the only other small delphinid species which could be potentially mismatched with striped dolphins is the short-beaked common dolphin (*Delphinus delphis*), which is rare or absent from our study area [19]. No common dolphins were observed in the photographed groups, and thus it is not unreasonable to assume that all small delphinids sightings were of *Stenella coeruleoalba*, i.e. the number of non-recognised common dolphins would be negligible. Additional sightings made during ‘off effort’ were considered as ‘secondary sightings’ and were not used in the abundance estimation analyses. Primary effort resumed when the plane rejoined the trackline where it had been left.

### Data analysis

Abundance was estimated using both conventional distance sampling or CDS [15] and multiple covariate distance sampling or MCDS [20]. The latter incorporates covariates, in addition to perpendicular distance, in the estimation of a detection function.

In CDS, animal abundance in each stratum is estimated by:
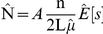
where, for each stratum, *A* is the area, L is the total search effort, *n* is the number of primary sightings, 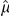 is the estimated effective strip half-width (*esw*) and 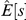 is the estimate of mean group size.

A variance estimate for 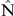 is obtained by combining the variance estimates of the three components, encounter rate, detection function and group size, using the delta method (eqn. 3.68 in [15]). The encounter rate variance is obtained using the R2 estimator of Fewster *et al.* [21]. Confidence intervals (CI) are lognormal confidence intervals based on equations 3.71–3.74 in [15], except that the normal distribution percentile is replaced with a *t*-distribution percentile, where the degrees of freedom are based on a method due to Satterthwaite [22].

In MCDS, covariates other than perpendicular distance are included in the detection function and hence the *esw* becomes a function of the covariates, *z*. Abundance is estimated using a Horvitz-Thompson-like estimator:
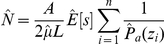
where 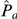 = estimated probability of detecting the ith object within w of the transect line and *z* = covariates.

The variance of this Horvitz-Thompson-like estimator is obtained using formulae described in Marques *et al.* [20] and lognormal CI are obtained as for CDS.

The minimum value of the Akaike Information Criterion or AIC [15], [23] was used to choose between models and select which covariates to include in the detection function.

A primary assumption of line transect distance sampling is that all animals on the trackline are detected [15]. This assumption may be violated for two main reasons: (1) the animals are underwater as the plane passes and are thus unavailable to be seen (known as availability bias) and (2) observers miss animals that were at the surface for one reason or another (known as perception bias). Both of these result in negatively biased abundance estimates, unless corrected for.

Limitations of the aircraft precluded the collection of data during the survey to estimate these biases, although methods exist [24].

A power analysis, using the software Trend [25], was performed to explore the 80% power of the summer survey results to detect changes in abundance over time. The following parameters were selected: a significance level α = 0.05; a 1-tailed test; a linear model of rate of change; the CV proportional to the square root of the abundance estimate; and a standard normal distribution. The summer CV of 17.2% was chosen.

### Striped dolphins

To fit the detection functions, 5% of the longest perpendicular distances were removed [15], leaving 106 out of 111 sightings for the winter and 260 of the 274 sightings for the summer; the truncation distances resulted in 365 m and 512 m for winter and summer, respectively.

**Figure 2:**
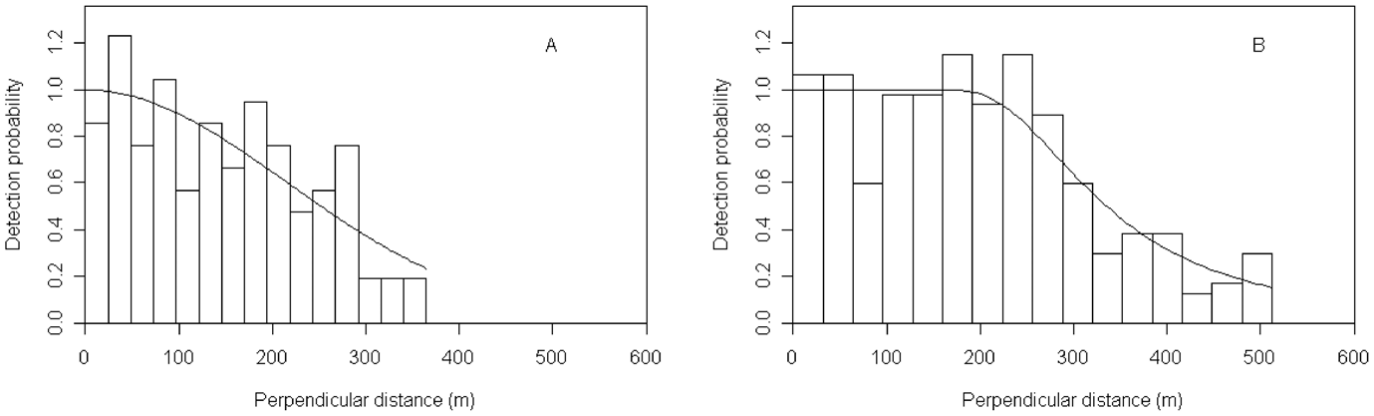
Striped dolphins' winter (A) and summer (B) detection functions.

On the basis of AIC, it was decided that for the striped dolphins CDS analysis, sightings should be pooled over all strata to fit a single detection function rather than fitting a separate detection function for each strata; a half normal form with no adjustment terms was chosen for the winter (Fig. 2A), and a hazard rate form with no adjustment terms was chosen on the basis of AIC for the summer (Fig. 2B). Estimates of encounter rate and group size were provided for each stratum for both surveys.

Customarily, since larger groups are generally easier to see at greater distances than smaller groups, to account for size bias in recorded group size, estimated group size is usually obtained from a regression of the logarithm of group size against detection probability [15]. In the winter, however, the expected group size from the regression (truncating at 5%) was larger than the mean group size. Therefore, the mean size of groups within 300 m of the trackline was used (since even the small groups had been detected within this range). In the summer dataset, the size-bias regression estimates of group size were as expected (i.e. smaller than the mean group size) and used for the analysis.

### Fin whales

**Figure 3:**
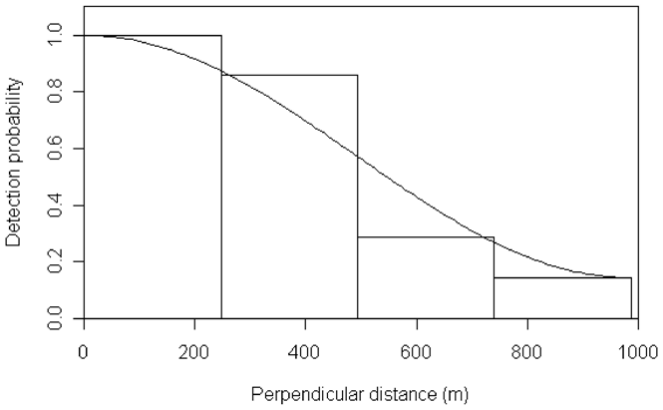
Fin whales' summer detection function.

The meagre number of fin whales sighted (primary sightings n = 16) during the summer survey precluded the use of the MCDS approach and no truncation was used (the largest perpendicular distance was 1000 m). While this is a rather small number of sightings, inspection of the fit of the detection function (Fig. 3) allowed us to conclude that this sample size was sufficient to develop an estimate of abundance. On the basis of AIC, a uniform key model with one cosine adjustment was chosen as the detection function.

## Results

**Figure 4:**
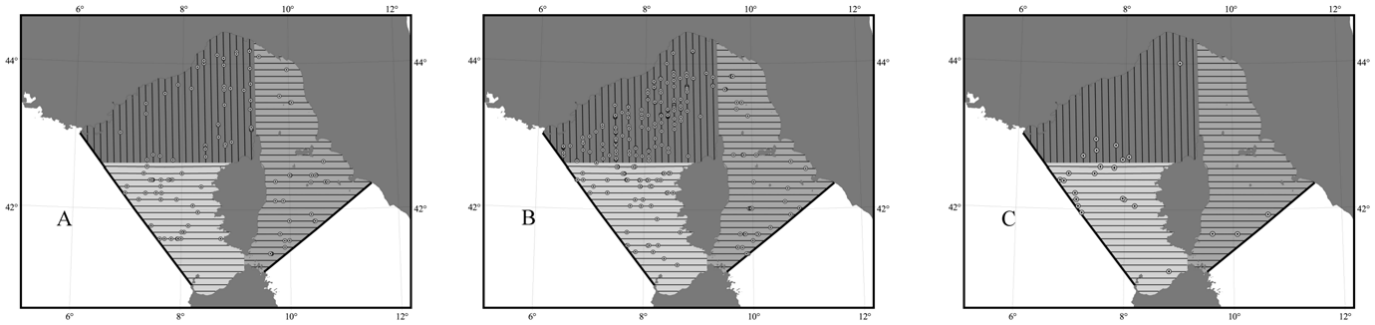
Maps of striped dolphins and fin whales sightings. Sightings of the two species for which sufficient sightings were made to estimate abundance: fin whales (summer only (A)) and striped dolphins (winter (B) and summer (C)).

**Figure 5:**
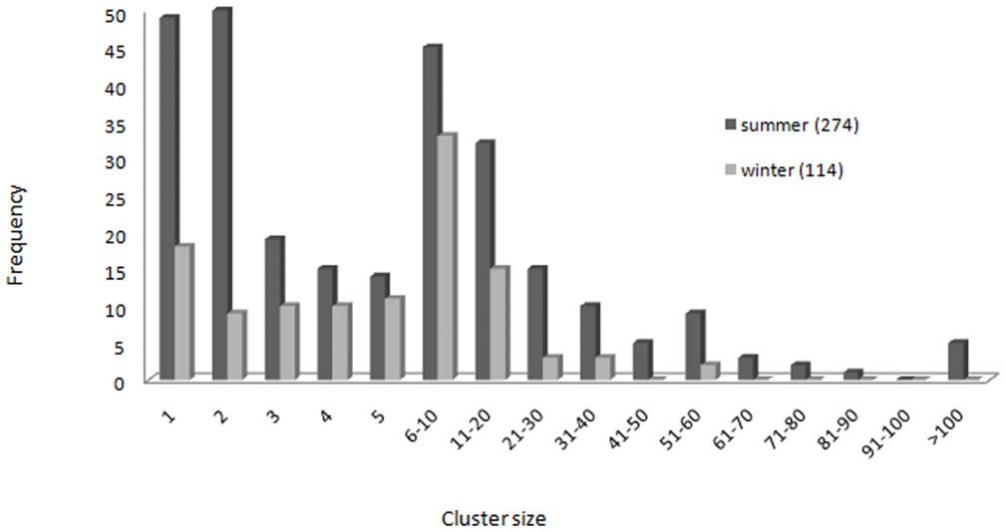
Winter and summer group sizes of striped dolphins.

Five cetacean species were sighted during the winter survey (Stenella coeruleoalba, Tursiops truncatus, Ziphius cavirostris, Physeter macrocephalus, Balaenoptera physalus) and seven (Sc, Tt, Zc, Pm, Bp, Grampus griseus and Globicephala melas) during the summer one. The total sightings (primary and secondary) made by survey and subdivided by each stratum are summarised in Table 1; the sightings of the two species for which sufficient sightings were made to estimate abundance - fin whales and striped dolphins - are plotted in Fig. 4 A,B,C. Group sizes of striped dolphins varied considerably during the two surveys, with larger groups sighted during the summer period (see Fig. 5).

**Table 1:** Total sightings data by season and stratum.

	Area	Species	Sightings		Group size		
			groups	individuals	mean	range	SD
Winter 2009	Stratum A (30 907 km^2^)	Striped dolphin	31	227	7.32	1–39	6.13
	k = 34	Bottlenose dolphin	6	18	3	1–6	1.788
	L = 2932.6 km	Cuvier's beaked whale	1	1	-	-	-
	Stratum B (23 208 km^2^)	Striped dolphin	34	234	6.88	1–27	4.86
	k = 20						
	L = 2273.5 km	Sperm whale	1	1	-	-	-
	Stratum C (34 153 km^2^)	Striped dolphin	49	447	9.12	1–57	7.08
	k = 22	Bottlenose dolphin	1	1	-	-	-
	L = 2938.3 km	Fin whale	1	1	-	-	-
	Total	Striped dolphin	114	908	7.96	1–57	6.44
	Area = 88 267 km2	Bottlenose dolphin	7	19	2.71	1–6	5.8
	K = 76	Cuvier's beaked whale	1	1	-	-	-
	L = 8144.4 km	Fin whale	1	1	-	-	-
		Sperm whale	1	1	-	-	-
Summer 2009	Stratum A (30 907 km^2^)	Striped dolphin	45	672	14.93	1–170	32.35
	k = 34	Bottlenose dolphin	5	22	4.4	1–8	3.05
	L = 3033.3 km	Fin whale	3	3	1	-	0
		Risso's dolphin	3	32	10.67	5–19	7.37
		Cuvier's beaked whale	2	5	2.5	2–3	0.71
	Stratum B (23 208 km^2^)	Striped dolphin	58	990	17.07	1–150	28.33
	k = 20	Fin whale	14	16	1.14	1–3	0.53
	L = 2264.7 km	Sperm whale	5	6	1.2	1–2	0.45
		Bottlenose dolphin	3	5	1.67	1–2	0.58
	Stratum C (34 153 km^2^)	Striped dolphin	171	2099	12.27	1–120	18.35
	k = 25	Fin whale	7	7	1	-	0
	L = 3148.8 km	Long-finned pilot whale	5	49	9.8	6–14	3.03
		Cuvier's beaked whale	2	5	2.5	2–3	0.71
		Risso's dolphin	1	2	-	-	-
	Total	Striped dolphin	274	3761	13.73	1–170	23.45
	Area = 88 267 km^2^	Fin whale	24	26	1.08	1–3	0.41
	K = 79	Bottlenose dolphin	8	27	3.37	1–8	2.72
	L = 8446.8 km	Long-finned pilot whale	5	49	9.8	6–14	3.03
		Sperm whale	5	6	1.2	1–2	0.45
		Risso's dolphin	4	34	8.5	2–19	7.42
		Cuvier's beaked whale	4	10	2.5	2–3	0.58

### Abundance and density estimates

#### Winter survey (11–31 January; 18–22 February)

The survey had to be carried out in two parts due to poor weather conditions and aircraft maintenance. A total of 8144 km (92%) of the planned trackline effort was completed. Four tracks in the northwestern corner of Stratum C could not be surveyed because of a French military no fly zone. Only for striped dolphins were there sufficient sightings to estimate abundance. Three sightings had a missing declination angle, leaving 111 primary sightings for the analysis.

The striped dolphins CDS estimate was 18 967 animals (CV = 22%; 95% CI 12 359–29 107). The additional covariates considered for inclusion in the MCDS model were observer, initial cue, cloud cover, glare, sea state and group size. On the basis of AIC, only observer (in addition to perpendicular distance) was included in the detection function. The detection function was estimated using data from all strata and again, the mean sizes of groups within 300 m were used. The resultant MCDS estimate was 19 462 (CV = 20.9%; 95% CI 12 939–29 273) with an estimated density of 0.22 animals per km^2^ (CV = 21%).

The CDS and MCDS estimates of total abundance were not substantially different, but on the basis of AIC the latter is preferred and is presented in Table 2.

**Table 2:** Abundance estimates for striped dolphins and fin whales from the 2009 winter and summer surveys.

Stratum	*L*	*n*	E[s] (%CV)	esw	*D (%CV)*	*N (%CV)*	95% CI(N)
**Striped (winter 2009)**							
A	2932.6	28	7.42 (25.8)	238.5 (6.2)	0.1486 (39.4)	4593 (39.4)	2150–9811
B	2273.5	31	6.76 (15.4)	238.5 (6.2)	0.1932 (34.2)	4484 (34.2)	2278–8829
C	2938.3	47	9.07 (20.1)	238.5 (6.2)	0.3041 (30.4)	10 385 (30.4)	5728–18 829
**Total**	**8144.5**	**106**			**0.2205 (20.9)**	**19 462 (20.9)**	**12 939–29 273**
**Striped (summer 2009)**							
A	3033.3	43	9.29 (27.9)	354.1 (5.4)	0.1859 (38.1)	5746 (38.1)	2761–11955
B	2264.8	53	12.05 (23.8)	354.1 (5.4)	0.3982 (34.4)	9241 (34.4)	4735–18034
C	3148.8	164	9.36 (13.1)	354.1 (5.4)	0.6881 (22.0)	23 501 (22.0)	15 217–36 294
**Total**	**8446.9**	**260**			**0.4360 (17.2)**	**38 488 (17.2)**	**27 447–53 968**
**Fin (summer 2009)**							
A	3033.3	2	1.00 (0.0)	564.6 (12.7)	0.00058 (70.7)	18 (70.7)	5–66
B	2264.8	8	1.00 (0.0)	564.6 (12.7)	0.00313 (32.6)	73 (32.6)	38–140
C	3148.8	6	1.00 (0.0)	564.6 (12.7)	0.00169 (46.4)	58 (46.4)	23–142
**Total**	**8446.9**	**16**			**0.00168 (27.4)**	**148 (27.4)**	**87–254**

*L* = km surveyed on effort. *n* = number of primary sightings used in the analysis. E[s] = estimated mean group size (%CV). *esw* = effective strip half width in m (%CV). *D* = density (animals per km^−2^) (%CV). *N* = estimated abundance (%CV). 95% CI (N). CV = coefficient of variation and CI = confidence interval.

#### Summer survey (21 July–2 August)

A total of 8494 km (97%) of the planned trackline effort was completed. There were sufficient primary sightings to estimate the abundance of striped dolphins and fin whales. Six striped dolphin sightings had either missing angle and/or group size leaving 274 primary sightings for the analysis. Of the 24 fin whale sightings, 16 were primary sightings - all of single animals - and thus used in the analysis.

The striped dolphins CDS estimate of abundance was 38 488 animals (CV = 17%; 95% CI 27 447–53 968) with a density of 0.44 animals per km^2^ (CV = 17%). For the MCDS analysis, AIC suggested that group size should be included in the detection function and the resultant estimate was 39 363 animals (CV = 16%; 95% CI 29 437–55 875). Note that although AIC suggests that the MCDS estimate should be preferred, with group size as a covariate estimates are not available by stratum [26], therefore the CDS estimates are given in Table 2.

The CDS estimated overall abundance of fin whales was 148 animals (CV = 27%; 95% CI 87–254), with a density of 0.00168 individuals km^−2^ (CV = 27%).

## Discussion

This study provides the first robust estimates of abundance and density of striped dolphins and fin whales for the whole Pelagos Sanctuary, as well as the first estimate of striped dolphins anywhere in the Mediterranean in the winter. These estimates are of numbers of animals within the area surveyed at the times of the particular year and season they were undertaken.

The abundance estimates provided in this paper are underestimates in that they have not yet been corrected for availability or perception bias; it may be possible to collect data in the future that will allow for such correction. To give a qualitative idea of the possible levels of bias, it is thought that perception bias for large whales, such as the fin whale is small: e.g. Heide-Jørgensen et al. [24] estimated a factor of around 0.86 for fin whales from an aerial survey off West Greenland using a different aeroplane – they did not provide an estimate for availability bias. Similarly, Palka [27] suggests that perception bias will also be small for larger groups of dolphins (mean group sizes for the present surveys were 8 in winter and 14 in summer). Gomez de Segura et al. [28], provided an availability bias correction factor for striped dolphins of around 0.7.

Correction for such biases, whilst important in terms of estimates of absolute abundance, is not important for trend analyses (the estimates can be treated as indices of abundance), provided that it can be assumed that the levels of bias remain constant among surveys over time. We assume this has been the case with our winter and summer surveys, given that the same plane, observers and field protocols were used.

### Seasonal changes in density within the Sanctuary

For both species, abundance and density values within the Sanctuary were appreciably higher in the summer (Table 2); in the case of the fin whale, only one sighting was made in winter and thus no abundance estimate could be made, but clearly the number of fin whales present was small. For striped dolphins the estimates are significantly higher (almost double) in summer.

These findings are in accord with the oceanographic information that shows a rich biomass in the Sanctuary in the summer, especially the western Ligurian Sea [1], [29], when compared to winter. Other authors have also found reduced densities of fin and striped dolphins within the region in winter, although the latter are found in considerable numbers, even in the winter, presumably reflecting *inter alia* the availability of their respective prey [30]–[33].

Our winter results confirm that fin whales tend to use the Sanctuary region seasonally [34]–[36], migrating elsewhere during the other seasons [37], [38], with acoustic data from the area revealing the presence of singing fin whales in autumn [39].

### Within season distribution within the Sanctuary

These surveys also provide information on within season density/distribution differences in the Sanctuary area (Table 2). In both winter and summer, striped dolphin density was by far the highest in Stratum C. The densities in Strata A and B were similar in winter but much higher in Stratum B in summer. This non-homogenous distribution within the Sanctuary probably reflects both dynamic (upwelling currents) and physiographic variables and their effect on striped dolphin prey, temporally and geographically [33], [40].

With respect to fin whales in summer, the density in Stratum B was almost double that in Stratum C, while density was very low in Stratum A. These results are in agreement with and quantify the findings of other authors [32], [33], [40] who have noted that fin whales concentrate in the deeper western offshore waters of the Sanctuary (and well beyond its borders, [41]).

### Conservation status

While it is valuable to compare density and abundance estimates from the present summer surveys with those obtained previously to consider status, a number of caveats must be made. The first is that density estimates obtained using analytical methods and/or using data from other platforms (e.g. vessels) are rarely strictly comparable, especially if they are uncorrected estimates. Most importantly, it is clear from the distribution of sightings (see Fig. 4) and information from previous surveys (see above) that the Pelagos Sanctuary does not encompass the full range of either species, even at the peak period of summer. This, along with the fact that population structure is poorly understood within the Mediterranean, means that differences in density and/or abundance may reflect interannual changes in distribution, rather than changes in population abundance. If the objective is to investigate population status and assess population level threats, rather than simply occurrence within the Sanctuary, then it is essential that the full ranges of the populations concerned are covered, at least periodically [14], [18]. It is for this reason that the ACCOBAMS Scientific Committee is recommending a basinwide synoptic survey [42].

That being said, such comparisons may allow tentative qualitative inferences on changes in status over time, suggesting testable hypotheses for future studies.

### Fin whales

A simple comparison of our 2009 results (or those from a shipboard survey carried out in 2008 – [43]) with published information from past shipboard surveys from either the whole Sanctuary area or parts of it [44], [45] – and see Appendix S1) suggests an appreciable decrease (perhaps by a factor of six) in the summer density and abundance of fin whales in the Pelagos Sanctuary area since the early 1990s. Although, as noted above, these data are not strictly and quantitatively comparable - given the different survey methods (aerial vs. ship-based) and the different portions of the Pelagos Sanctuary covered by the various surveys - we do believe that the apparent decline is sufficient to warrant some caution. While it is not appropriate to directly use correction factors from other aerial surveys and other species to account for availability bias, in addition to the example of Heide-Jørgensen et al. [24], the approach used by Andriolo *et al.* [46] led to a correction factor for humpback whales (*Megaptera novaeangliae*) off Brazil of the order of around 0.7. If such values are correct, then this would support the inferred decline. While this observed decrease of fin whales in the Pelagos Sanctuary may be due to whales relocating elsewhere within the Mediterranean, their decrease in prime fin whale habitat must be addressed with precaution, and a population decline in the Mediterranean cannot be discounted at this time. It is important to investigate this further, since if it is a true population decline, then serious conservation actions are required. The best known human induced cause of direct mortality of fin whales in the Mediterranean is collisions with ships, although there are insufficient data on both whale abundance and numbers of deaths to determine whether this represents a population level threat [5]. Vessel traffic in summer within the Sanctuary is high and has been increasing – this may result in increased collisions (especially from high speed ferries) or increased overall disturbance (including from whale-watching vessels – [47]), causing the whales to move elsewhere. Other potential issues relate to more indirect threats such as effects of chemical pollutants on reproduction and survivorship [48], effects of ocean acidification or climate change on prey [7] and the synergistic effects of some or all of these factors.

### Striped dolphins

By contrast with fin whales, the available information for striped dolphins suggests a qualitative slight increase in density/abundance since 1991 (same caveats discussed for fin whales regarding g(0) apply for striped dolphins as well), at least within the north-west portion of the Sanctuary (strata A and B) [44], [49], while striped dolphins' abundance seems to be rather stable since 2001 [45]. In the past, the most serious threat to Mediterranean striped dolphins was thought to be bycatch in pelagic driftnets, which was believed to be at unsustainable levels at least up to the early 1990s [50]. In addition, a major morbillivirus-related die off occurred from 1990–92 [see review in 4] although it was not possible to obtain accurate total numbers of mortality or to determine what the population level effects might be. For these reasons, it is possible that the Mediterranean striped dolphins had been depleted, perhaps considerably, by the mid-1990s.

Despite the lack of firm quantitative information, there are reasons to believe that some recovery may have occurred. For example, the European Union established a driftnet ban in 2002 (Council Regulation n° 1239/98). It is clear that compliance has not been perfect, even within the Sanctuary [51], [52] and, according with the general illegality, both Italy and France have been called upon by the European Commission to ensure compliance with the EU rules. In 2009, Italy was also asked to return the Community fund used for the recovery plan (Piano Spadare), while France was condemned for the lack of control over the use of driftnets. Given this, although no good recent bycatch mortality estimates exist [43], it seems likely that such mortality is lower now than in the past, and driftnetters and thonaille (the French driftnet) are both disappearing from the Pelagos Sanctuary [53]. Indirect evidence for the mortality reduction comes from the Italian stranding network data (http://mammiferimarini.unipv.it/spiaggiamenti.php). However, it is essential that bycatch monitoring is improved to enable robust estimates to be obtained, in order to examine population level effects. Although, more recent morbillivirus episodes have occurred [54], they do not seem to have resulted in mortality at the same scale as previously. Thus, the limited available information does not rule out the idea that striped dolphins may be increasing, but it is essential that quantitative data on threats, population structure, abundance and trends be collected to allow a thorough evaluation of the status of striped dolphins, within and outside the Sanctuary.

### Other cetacean species

During both aerial surveys, other cetacean species were observed, even if the small sample size did not allow any abundance and density estimate. The data collected during the aerial surveillance provided insights on distribution and occurrence within the whole Sanctuary area, will be used for habitat modelling and will be pulled for future estimates, once additional data collected during further aerial surveys will be available.

### Conclusions

The programme thus far has illustrated the value of aerial surveys for providing robust estimates of cetaceans' abundance (and indices of abundance) and density in all seasons of the year. In addition, during these surveys other marine megafauna has been observed, with species such as the loggerhead turtle (*Caretta caretta*), the giant devil ray (*Mobula mobular*) and the basking shark (*Cetorhinus maximus*), which have been listed in the Annex II, list of endangered or threatened species of the Protocol concerning Specially Protected Areas and Biological Diversity in the Mediterranean (SPA/BD) within the Barcelona Convention, and therefore need specific conservation measures.

The advantages of this approach over more traditional ship-based surveys can be summarised as follows:

1. greater efficiency in terms of coverage (track length×effective search width);
2. ability to take advantage of short periods of good weather (especially important in winter);
3. much less uncertainty in distance measurements and group size estimates, critical components of the overall uncertainty in abundance estimates using distance-based methods;
4. little or no problem with responsive movement or avoidance due to the platform at the survey height used (this may cause significant bias with vessel surveys for some species – see [55], [56]).

Of course, there are circumstances where vessels are more appropriate (e.g. where landing facilities and aircraft endurance make coverage of offshore areas impossible, where collection of biopsy samples and photo-identification photographs is important); Table 3 presents a simple comparison between the two methods, obviously local factors and costs need to be borne in mind when making decisions.

**Table 3:** Simple comparison of strengths of vessel and aircraft survey platforms for the Mediterranean Sea.

VESSEL	AIRCRAFT
**Area covered**	
Small vessels: coastal watersLarge vessels: high seas	Generally limited to coastal waters but depends on fuel capacity/endurance and availability of airports
Travel speed around 10 knots limits area coverage with time	Travel speed around 100 knots means around 10 times greater search distance with time
Poor for areas with complex coastlines and small islands	Deals with complex coastlines and small islands well
**Species**	
Relates to area that can be covered and behaviour, but in principle all species either visually or acoustically	Better suited to the non long-divers given speed of platform; not good for high seas species given endurance limitations
Need to account for potential responsive movement	Responsive movement not a problem
School size estimation for some species can be difficult	Generally easier to estimate school size
Generally poor for estimating other megafauna	Good for other megafauna (e.g. sea turtle, giant devil ray, sharks, tuna) at least in the Mediterranean Sea
**Environmental conditions**	
Cannot operate in ‘unacceptable’ conditions (these will depend on species) – swell can be a major problem	Cannot operate in ‘unacceptable’ conditions (these will depend on species) – swell less of a problem
Given speed limitations, relatively poor use of good weather windows	Efficient use of good weather windows (higher survey speed, ability to move to good weather areas quickly)
**Data collection**	
Measurement of key parameters, especially distance, and to a lesser extent angle, is problematic	Measurement of perpendicular distance easier and better
Estimation of g(0) using double platform methods well established and space on board usually not a problem	Difficult to use double platform methods in smaller planes (for some species ‘circle back’ works [60]) but possible in larger planes
Allows collection of additional data: acoustic, environmental, photo-identification data	Collection of additional data difficult or impossible
Usually can incorporate more scientists	Limited number of scientists
**Cost**	
More expensive than aerial surveys but:can operate on high seas;can collect additional data.	More cost-effective where they can operate and better able to take advantage of good conditions when they are scarce (both geographically and seasonally)

Long-term monitoring of population abundance to inform conservation measures is essential, but often does not occur [14]. In fact, the EU Habitats Directive requires Member States to monitor natural habitats and species of wild fauna and flora of Community interest and to report every six years on whether their conservation status is favourable and on the implementation of measures taken to ensure this (Council Directive 92/43/EEC). In addition, the Marine Strategy Framework Directive (MSFD) states that “Member States shall establish and implement coordinated monitoring programmes for the ongoing assessment of the environmental status of their marine waters”. This assessment is based on three criteria: species distribution (distributional range and distributional pattern within this rage), population size (population abundance) and population condition (demographic characteristics) (COM_DEC 2010/477/EU). Similarly, monitoring of human activities that may affect the status of populations of interest is an essential component of conservation and management [57]. Without both of these and effective conservation measures, the value of a Sanctuary must be questioned.

This work represents a first step for the evaluation of management and conservation effort within the Pelagos Sanctuary, in compliance with the European Union regulations mentioned above. In addition, it may be considered as an example for the management of high seas SPAMIs, particularly considering the recent effort to “identify potential sites for the creation of Specially Protected Areas of Mediterranean Importance (SPAMIs) in the open seas, including the deep sea” carried out by the Regional Activity Centre for Special Protected Areas (RAC/SPA) under the auspices of the United Nation Environmental Programme - Mediterranean Action Plan (UNEP-MAP).

The current commitment by the Italian Ministry of the Environment is providing a baseline for systematic monitoring programmes that will eventually be able to assess population trends and to quantify the impact of human threats and potential negative effects on the Pelagos Sanctuary striped dolphins and fin whales. Further surveys using the same protocols and covering other areas have been carried out in 2010. According to the power analysis, the timeframe to detect a 2% rate of annual decline would be 18 years, 10 years for a 5% rate of change, 7 for 10% and 6 for 15%. As new information becomes available on population structure and the full range of populations, monitoring programmes following the power analyses results will be planned, determining the optimum frequency and survey design to be able to detect trends in abundance [58], [59] and inform policy makers on the most appropriate measures to undertake.

In conclusion, taking into consideration that in recent IUCN Red List assessments the Mediterranean striped dolphin and fin whale sub-populations have been suggested as Vulnerable [3], the results presented in this paper strongly support the need for long-term monitoring and for appropriate conservation measures throughout the whole Basin. The information obtained also suggest an expansion of the survey area, covering the whole north-western Mediterranean Sea - including the Pelagos Sanctuary, the Gulf of Lions and the Balearic Basin - thus greatly facilitating the planning for the urgently needed synoptic ACCOBAMS basinwide survey, illustrating the value of aerial surveys for cetacean and other marine species of conservation concern to the effort.

## Supporting Information

Appendix S1 **Summary of abundance estimates for striped dolphins and fin whales in the western Mediterranean Sea.**
(DOC)
