# Supplementary material for: Monitoring Winter and Summer Abundance of Cetaceans in the Pelagos Sanctuary (Northwestern Mediterranean Sea) Through Aerial Surveys
Source: PLoS One. 2011 Jul 29;6(7):e22878. doi: 10.1371/journal.pone.0022878 (PMC3146501; doi:10.1371/journal.pone.0022878)
Supplement: Appendix S1 — Summary of abundance estimates for striped dolphins and fin whales in the western Mediterranean Sea. (DOC) [file pone.0022878.s001.doc]

**APPENDIX S1**  – Summary of abundance estimates for striped dolphins and fin whales in the western Mediterranean Sea.

| **Geographic Area** | **Species** | **Method** | **Year of Estimate** | **Estimated Animals** | **95% C.I.** | **Density** | **%CV (D)** | **Source** |
| --- | --- | --- | --- | --- | --- | --- | --- | --- |
| Western Mediterranean (excluding the Tyrrhenian Sea) | Striped dolphin | Shipboard | 1991 | 117,880 | 68,379-214,800 | 0.19857 (summer) | 33 (summer) | [S1] |
| Liguro-Provençal Basin | Striped dolphin | Shipboard | 1991 | 42,604 | 24,962-72,716 | 0.24 (summer) | 26.0 (summer) | [S2] |
| Ligurian Sea | Striped dolphin | Shipboard | 1991 | 14,003 | 6,305-31,101 | 0.30 (summer) | 35.0 (summer) | [S2] |
| Balearic Sea | Striped dolphin | Shipboard | 1991 | 5,826 | 2,193-15,476 | 0.09 (summer) | 36.0 (summer) | [S2] |
| Provençal Basin (Gulf of Lions) | Striped dolphin | Shipboard | 1991 | 30,774 | 17,433-54,323 | 0.23 (summer) | 25.0 (summer) | [S2] |
| South Balearic Area | Striped dolphin | Shipboard | 1991 | 18,810 | 8,825-35,940 | 0.08 (summer) | 34.0 (summer) | [S2] |
| Southwestern Mediterranean | Striped dolphin | Shipboard | 1991 | 39,963 | 18,206-87,721 | 0.12 (summer) | 38.0 (summer) | [S2] |
| Northwestern Mediterranean | Striped dolphin | Shipboard | 1991 | 48,098 | 29,388-78,721 | 0.40 (summer) | 0.24 (summer) | [S2] |
| Alboran Sea | Striped dolphin | Shipboard | 1992 | 17,728 | 9,507-33,059 | 0.20 (summer) | 33 (summer) | [S2] |
| Western Pelagos Sanctuary | Striped dolphin | Shipboard | 1996 | 28,385 | 20,058-37,820 | 0,56 (summer) | 16,4 (summer) | [S3] |
| Central coast of Spain | Striped dolphin | Aerial | 2001-2003 | 15,778 | 10,940-22,756 | 0.489 (mean value) | 18.8 (mean value) | [S4] |
| Central coast of Spain | Striped dolphin | Aerial | 2002-2003 | 18,721 | 10,373-33,793 | 0.580 (winter) | 30.8 (winter) | [S4] |
| Central coast of Spain | Striped dolphin | Aerial | 2001-2002 | 16,859 | 10,499-27,072 | 0.522 (summer) | 24.5 (summer) | [S4] |
| South Tyrrhenian Sea | Striped dolphin | Shipboard | 2002 | 3,529 | 1,506-8,269 | 0.26 (summer) | 42 (summer) | [S5] |
| South Tyrrhenian Sea | Striped dolphin | Shipboard | 2003 | 4,030 | 2,239–7,253 | 0.30 (summer) | 30 (summer) | [S5] |
| Entire Pelagos Sanctuary | Striped dolphin | Shipboard | 2001 | 37,526 | 22,450–62,856 | 0.52 (summer) | 26.2 (summer) | [S6] |
| Western Ligurian Sea and the offshore waters of western Corsica | Striped dolphin | Shipboard | 1992 | 25,614 | 15,377-42,658 | 0.4396 (summer) | 25.34 (summer) | [S7] |
| Ligurian Sea | Striped dolphin | Shipboard | 2010 | NA | NA | 0.87 (summer)  0.37 (winter) | 15.5 (summer)  21.7 (winter) | [S8] |
| Corsican-Ligurian-Provençal Basin | Striped dolphin | Shipboard | 2008 | 13,232 | 6,640-26,368 | 0.23 (summer) | 35.55 (summer) | [S9] |
| Western Mediterranean Sea | Fin whale | Shipboard | 1991 | 3,583 | 2,130-6,027 | 0.02408 (summer) | 27.0 (summer) | [S10] |
| Western Ligurian Sea and the offshore waters of western Corsica | Fin whale | Shipboard | 1992 | 901 | 591-1,347 | 0.0155 (summer) | 21.77 (summer) | [S7] |
| Entire Pelagos Sanctuary | Fin whale | Shipboard | 2001 | 715 | 421-1,215 | 0.097 (summer) | 31.2 (summer) | [S6] |
| Ligurian Sea | Fin whale | Shipboard | 2001-2004 | NA | NA | 0.014 (summer) | 19.2 (summer) | [S8] |
| Ligurian Sea | Fin whale | Shipboard | 2001-2004 | NA | NA | 0.002 (winter) | 46.3 (winter) | [S8] |

**REFERENCES**

S1. Forcada J, Aguilar A, Hammond PS, Pastor X, Aguilar R (1994) Distribution and numbers of striped dolphins in the western Mediterranean Sea after the 1990 epizootic outbreak. Mar Mammal Sci 10: 137-150.

S2. Forcada J, Hammond PS (1998) Geographical variation in density and numbers of striped and common dolphins of the western Mediterranean. J Sea Res 39: 313-325.

S3. Gannier A (1998) Les cétacés de Méditerranée nord-occidentale: nouveaux résultats sur leur distribution, la structure de leur peuplement et l’abondance relative des différentes espèces. Mésogée 56: 3-19.

S4. Gomez de Segura A, Crespo EA, Pedraza SN, Hammond PS, Raga JA (2006) Abundance of small cetaceans in the waters of the central Spanish Mediterranean. Mar Biol 150: 149-160.

S5. Fortuna C, Canese S, Giusti M, Revelli E, Consoli P, et al. (2007) An insight into the status of striped dolphins (*Stenella coeureoalba*) of the southern-Tyrrhenian sea. J Mar Biol Ass U K 87: 1321-1326.

S6. Gannier A (2006) Le peuplement estival de cetaces dans le Sanctuaire Marin Pelagos (Mediterranee nord-occidentale): distribution et abondance. Mammalia 70: 17-27.

S7. Forcada J, Notarbartolo di Sciara G, Fabbri F (1995) Abundance of fin whales and striped dolphins summering in the Corso-Ligurian Basin. Mammalia 59: 127-140.

S8. Laran S, Joiris C, Gannier A, Kenney RD (2010) Seasonal estimates of densities and predation rates of cetaceans in the Ligurian Sea, northwestern Mediterranean Sea: an initial examination. J Cetacean Res Manage 11: 31–40.

S9. Lauriano G, Panigada S, Canneri R, Manca Zeichen M, Notarbartolo di Sciara G (2011) Abundance estimate of striped dolphins (Stenella coeruleoalba) in the Pelagos Sanctuary (NW Mediterranean Sea) by means of line transect survey. J Cetacean Res Manage 11: 279–283.

S10. Forcada J, Aguilar A, Hammond PS, Pastor X, Aguilar R (1996) Distribution and abundance of fin whales in the Western Mediterranean Sea during the summer. J Zool 238: 23-34.
